# The information trail

**DOI:** 10.7554/eLife.16019

**Published:** 2016-04-12

**Authors:** Carol Berman

**Affiliations:** Department of Anthropology and the Graduate Program in Evolutionary Biology, Ecology and Behavior, State University of New York at Buffalo, Buffalo, United Statescberman@buffalo.edu

**Keywords:** social information, social networks, chacma baboon, papio ursinus, network based diffusion analysis, Other

## Abstract

The ability of a wild baboon to acquire and exploit social information depends on its individual characteristics and its position within various social networks.

**Related research article** Carter AJ, Ticó MT, Cowlishaw G. 2016. Sequential phenotypic constraints on social information use in wild baboons. *eLife* **5**:e13125. doi: 10.7554/eLife.13125

**Image** Small clusters of baboons leave the cliffs where they sleep to forage for food at dawn

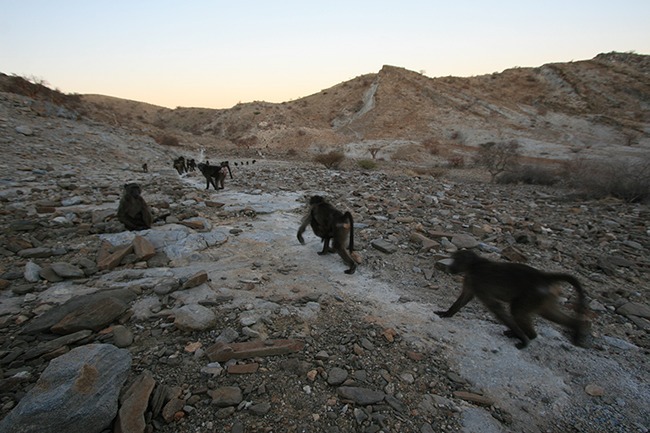

Organisms need information about their environment in order to survive and reproduce. Many organisms are able to learn about their environment directly by observing it and interacting with it: this is called "personal learning". However, some species, particularly those that live in social groups, can also learn indirectly by observing and interacting with individuals who already have knowledge. There are plenty of examples of such "social learning" in nature: for instance, many species are able evade predators because they can sense alarm signals produced by other members of their own or other species; this means that they can avoid the predator without ever seeing, hearing or smelling it (Fichtel, 2012). In other cases, females choose territories for breeding based on the appearance and vigor of the males holding the territory, rather than on the quality of the territory itself; this is because strong, attractive males tend to have high-quality territories. Finally, many animals that forage or roost near one another learn about where they can find food by observing the behavior of their companions (Valone and Templeton, 2002).

Social learning is often more efficient and less risky than personal learning, although the information gained may be outdated or unreliable (Giraldeau et al., 2002). However, the efficiency of social learning means that social information can potentially spread rapidly through a social group, and then be transmitted to other groups by migrants, as well as being passed on from generation to generation. As such social learning forms the cornerstone of both animal and human cultural behavior. Understanding how it works in groups of social animals will help us to understand the evolution of both animal and human culture (Castro and Toro, 2004).

Several previous studies have addressed the question of how socially learned information and skills spread throughout social groups, particularly in socially-living non-human primates. These groups are generally organized around kinship relationships, such that individuals who are closely related to each other favor one another. Not surprisingly, researchers have found that information is often transmitted along family lines and among other close associates. In a classic study of Japanese macaques, a new way to process food was first invented by a juvenile female. It then spread to the juvenile's playmates (both of the same age and older) and their mothers, and then gradually to the rest of their extended families. Eventually, infants learned the skill from their mothers. Adult males, who occupy peripheral positions in the social network, were slow to learn (Kawai et al., 1992). Other studies have shown that additional factors, such as dominance rank (that is, position in the dominance hierarchy of the group), are crucial in determining how social information spreads (Drea and Wallen, 1999). However, it should be noted that some researchers have argued that the results of the study on Japanese macaques are better explained by personal learning (Galef, 1992).

Now, in eLife, Alecia Carter of Cambridge University and colleagues – Miquel Torrents Ticó and Guy Cowlishaw of the Institute of Zoology – have used new social network analysis techniques to explore how social information is transmitted in troops of wild baboons (Carter et al., 2016; Figure 1). In particular, they explored how individual characteristics, including cognitive, social, ecological and demographic factors, influenced social learning. Past studies typically looked only at final outcomes: for example, did an individual demonstrate a particular skill? In contrast, Carter et al. break the process down into three sequential stages – the acquisition of information, the application of information, and the exploitation of its benefits – and look at the factors that may limit or favor individuals at each of these three stages.

**Figure 1.**
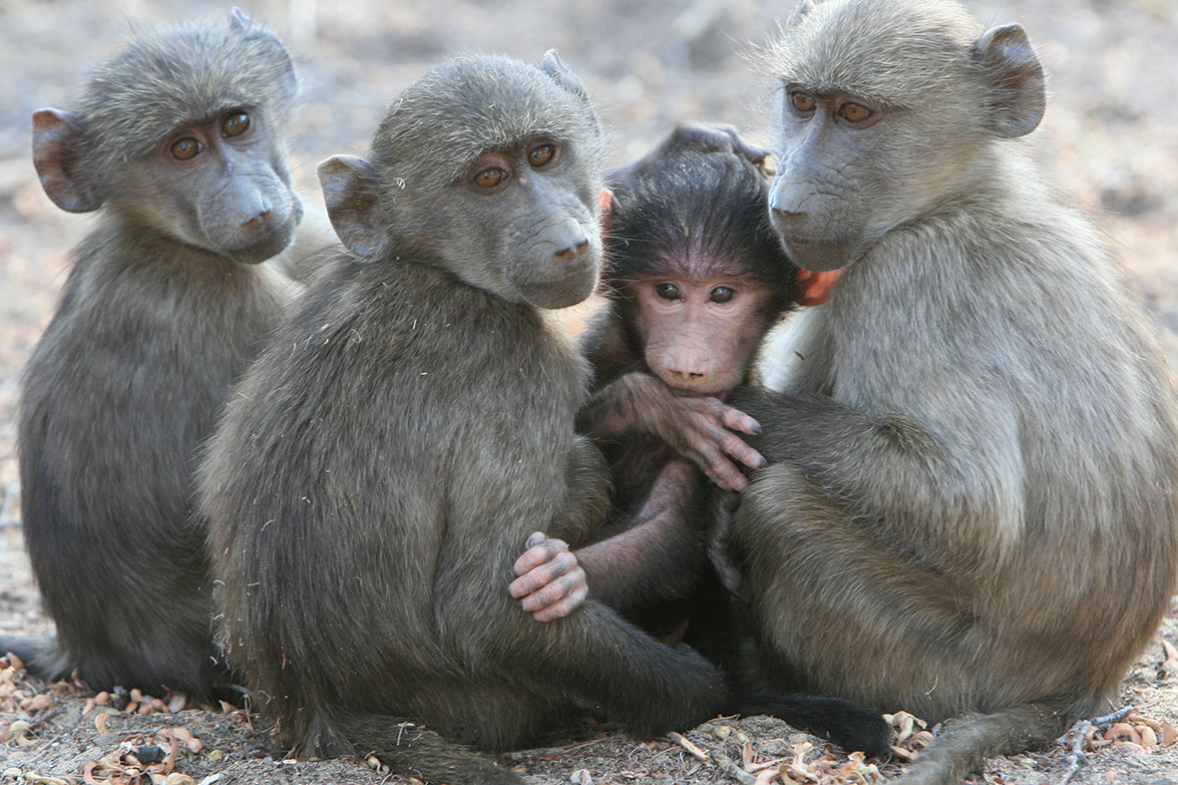
Wild chacma baboons in Tsaobis Nature Park, Namibia. Carter et al. found that close social ties between baboons increased the application and exploitation of socially acquired information about the location of food patches. *Photograph: Alecia Carter*

They surreptitiously placed small amounts of maize kernels (a favorite food of baboons) in the paths of two foraging troops of wild chacma baboons (*Papio ursinus*) in Tsaobis Nature Park, Namibia. As the baboons approached this food patch, Carter et al. recorded the identities of the baboons that found the food independently (and the order in which they did so) and the identities of those that found the food by watching a finder ingest it (information acquisition). They also recorded which baboons entered into the food patch (information application) and which baboons ate the food (exploitation of benefits). After the initial discovery of the food, nearly all the discoveries were made by social learning.

A troop of baboons at this site can contain as many as 55 baboons. Each individual baboon forms social bonds with other members of the group – some of these bonds are close, others are distant or some are even antagonistic. Researchers use social networks based on factors such as proximity, grooming and dominance to describe the overall structure of these bonds. Using a technique called order of acquisition diffusion analysis (Hoppitt et al., 2010), Carter et al. identified the particular social network that best accounted for their data. This network connected individuals who travelled together in clusters that were dispersed over 10 meters or less. Networks based on grooming and dominance were less able to predict the results of the observations, as were networks of a different size.

Carter et al. then explored whether individual characteristics – such as age, sex, rank, boldness and position within a particular social network – influenced the acquisition, application and exploitation of the social information about the food patches. Individuals that had central positions within the 10 meter proximity network were most likely to learn the food location socially. Having a central position in this network and also in the grooming network (that is, having many strong grooming relationships) facilitated entrance into the food patch (application), as did being male. Strong grooming ties, high dominance status, maleness and boldness facilitated actual feeding (exploitation). On average, less than 25% of baboons acquired the social information about the food and less than 5% exploited it.

The results show that is useful to break down the transmission of social information into three sequential stages, with each stage being governed by different individual and social attributes. Carter et al. suggest that when social information can be acquired by visual means alone, the transmission of information is limited only by an individual’s spatial relationships with other group members. However, the application and exploitation of information are both more likely to involve other attributes. In this study, the application of information also involved strong social ties, which suggests that baboons that were feeding tolerated their close grooming partners. Social ties also influenced the exploitation of information, and power relationships were particularly important, with dominant males and bold individuals being most likely to feed.

The precise characteristics and success rates identified by Carter, Ticó and Cowlishaw are likely to be specific to the conditions of their study. For example, success rates might be higher at all stages if the food patches were larger. The next challenge, therefore, is to further explore the relevant parameters and general principles that guide the transmission of social information under a range ecological, social and demographic conditions.
